# The pragmatic role of trust in young children’s interpretation of unfamiliar signals

**DOI:** 10.1371/journal.pone.0224648

**Published:** 2019-10-30

**Authors:** Olivier Mascaro, Dan Sperber

**Affiliations:** 1 Institute for Cognitive Sciences, CNRS UMR5304/Lyon 1 University, Bron, France; 2 Social Mind Center, Central European University, Budapest, Hungary; Harvard Medical School, UNITED STATES

## Abstract

What role does children’s trust in communication play in their acquisition of new meanings? To answer, we report two experimental studies (*N* = 81) testing how three- to four-year-olds interpret the meaning of a novel communicative device when it is used by a malevolent and potentially deceptive informant. Children participated in a hiding game in which they had to find a reward hidden in one of two boxes. In the initial phase of the experiments, a malevolent informant always indicated the location of the empty box using a novel communicative device, either a marker (Study 1), or an arrow (Study 2). During that phase, 3- and 4-year-olds learned to avoid the box indicated by the novel communicative device. In the second phase of the test, the malevolent informant was replaced by a benevolent one. Nevertheless, children did not change their search strategy, and they kept avoiding the box tagged by the novel communicative device as often as when it had been produced by the malevolent informant. These results suggest that during the initial phase, children (i) did not consider the possibility that the malevolent informant might intend to deceive them, and (ii) did not ignore the unfamiliar communicative signal or treat it as irrelevant. Instead, children relied on the unfamiliar communicative signal to locate the empty box’s location. These results suggest that children’s avoidance of the location indicated by an unfamiliar signal is not unambiguous evidence for distrust of such signal. We argue that children’s trust in ostensive communication is likely to extend to unfamiliar communicative means, and that this presumption of trustworthiness plays a central role in children’s acquisition of new meanings.

## Introduction

As David Lewis [1] among others argued, epistemic trust plays a crucial role in the acquisition of word meaning. If your interlocutor says, pointing to a flower you don’t know, “look at this gorgeous sobralia”, you might infer that the word “sobralia” refers to a kind of flower of which this is an exemplar. This pragmatic inference is only justified, however, if you trust your interlocutor to speak competently and honestly. Children, who acquire new words every day, must similarly trust their interlocutors to speak truly in order to infer the meaning of these words (and more generally of forms of communication they are not familiar with). Here we test how young children’s inferences about the meaning of a new non-verbal signal might be influenced by trust.

Philosophers and psychologists agree with common wisdom that young children are trusting [2–3]. But how trusting are they? If they were gullible to the point of unconditionally believing whatever they are told, they should remain helpless and dumbfounded when two informants provide incompatible testimonies. Recent work in developmental psychology has shown that such is not the case. When given conflicting testimonies by two informants, children trust more an informant who has been accurate before, is more confident, has better access to information, or is more benevolent [3–7]. Other studies have shown that children can disbelieve communicated information that conflicts with what they perceive or remember [8–10].

So, children are not trusting to the point of being wholly unable to choose among two conflicting sources of information. But what happens when there is a single informant whose testimony does not contradict the child’s own beliefs? In such a case, do children always trust the testimony or are they capable, as adults are, to sometimes disbelieve or just ignore it? What happens in particular if children are given strong evidence that the informant may be providing them with false information? In previous studies, it was found that three-year-olds (but not four-year-olds) told that an informant always lies nonetheless accepted his or her testimony, and that four-year-olds (but not five-year-old) told that an informant did not want to help them did likewise [11]. It has also been found that despite their capacity to treat the utterance of a single speaker as false [12], young pre-schoolers tend to persevere in trusting a single informant who has repeatedly misled them [11, 13–15].

Children are trusting to the point of failing to take into account repeated evidence that an informant is untrustworthy and likely to deceive them. Why? One hypothesis is that children understand communication as a helpful behavior [13, 16]. As argued by Grice [17] or Sperber and Wilson [18], by the very act of communicating, communicators present themselves as benevolent and competent, willing and able to provide true and relevant information to their audience. The very fact of being addressed by a communicator elicits in the audience an initial ‘stance of trust’ [19] that may be stronger in younger children. Actually, the acquisition of cultural knowledge would not be possible if children were unwilling to pay attention to and to take a trusting attitude towards forms of communication with which they have little or no familiarity.

Children learn words that they have never encountered before and they do so on the basis of little exposure [20–21]. These words are comprehended and used before becoming familiar. Similarly, children learn from demonstrations about unknown objects, which often involve a set of unfamiliar demonstration gestures. When engaging in this kind of learning, children display trust in what is conveyed to them by means of an unfamiliar communicative action [22–23]. Overall the data thus suggest that children have a general tendency to trust communication (or more specifically ‘ostensive communication’; see [24]) whoever communicates and whatever means of communication are being used.

According to a second (not mutually exclusive) hypothesis, young children are disposed to believe what is communicated to them because of a more specific trust in signals that they have repeatedly experienced to be reliable in the past (for discussions see [14, 25]). For example, in Palmquist, Burns, and Jaswal [26], three-year-old children saw one informant hide a sticker and another fail to pay attention. Then both informants indicated where they thought the sticker was, either by pointing or by using an unfamiliar signal. When both informants pointed, children didn’t trust one more than the other. When, on the other hand, both informants used unfamiliar signals, then children chose the location indicated by the more knowledgeable informant. One interpretation of these results is that the high level of trust elicited by familiar signals, such as pointing, can disrupt children’s capacity to track informants’ knowledge.

In short, when children are addressed by a single informant who uses familiar communicative means such as pointing or verbal testimony, they seem to be spontaneously trustful. The lack of vigilance that children evidence in these situations may have two sources: (i) a general disposition to treat communicated information as reliable (regardless of the communicative means employed), and (ii) a more specific disposition to treat as reliable signals that have proven to be reliable in the past.

Two studies, one by Couillard and Woodward [27], and one by Jaswal, Croft, Setia, and Cole [14] provide relevant evidence to test these two hypotheses. In Couillard and Woodward [27], preschoolers had to find a sticker hidden under one of two upside-down bowls. The experimenter misleadingly indicated the empty bowl either by pointing toward it or by placing a marker on it. Over the course of 10 trials, 3-year-olds were more likely to persevere and search under the empty bowl when the experimenter pointed to it than when she placed a marker on it. Similarly, in Jaswal et al., [14] 3-year-olds either heard an experimenter claim that a sticker was in one location when it was actually in another one, or they saw her place an arrow on the empty location. In both conditions, children initially tended to search the wrong location, but then those who were hearing the deceptive verbal testimony persevered, whereas those who saw the incorrect location being marked with an arrow quickly learned to search in the correct location. On the face of it, these results could be interpreted as evidence that when a misleading informant uses unfamiliar communicative means, young preschoolers are less trusting than when the misleading informant uses a familiar communicative means.

These results are, however, open to an alternative interpretation. When an informant’s communicative behavior has misled you, you might either withdraw your trust in the informant or still trust the informant but revise your interpretation of the signal. As the evidence shows, when the signal used—pointing or speech—is familiar, 3-year-olds do not learn anything from being repeatedly misled. They neither withdraw their trust nor reinterpret the signal. On the other hand, when the informant uses an unfamiliar signal, children do seem to learn. What do they learn? There are two possibilities 1) They might learn that the communicator is misinforming them and withdraw their trust; 2) they might maintain their trust and use this trust as a premise in inferring that the signal indicates not where the reward is, but where it is not.

We report two experimental studies that compare these two hypotheses. Three- and four-year-olds participated in a task in which they had to find an object hidden in one of two boxes. They were provided information by means of an unfamiliar signal, i.e. by the placement of a marker on one of the two boxes in Study 1, following [27–28], and by an arrow in Study 2, following [14].

During the initial phase of the study, a first informant, who was explicitly said to be mean and uncooperative, always indicated the empty box with the unfamiliar communicative device (i.e., placing the marker on the empty box, or pointing towards it with the arrow). We hypothesized that during this first phase, children would learn to avoid the location indicated by the unfamiliar communicative device (just like children had done in [14; 27]). In the second phase of the study, the mean informant was replaced by a different informant who was said to be nice and willing to help the children. This second phase was intended to provide evidence of the inferences children had drawn in the first phase of the study (where a first “mean” informant had repeatedly indicated the empty box with the unfamiliar communicative device).

A first possibility is that children had interpreted the unfamiliar communicative device as meaning that the box that it indicated contained the hidden object and, given that this box turned out one trial after the other to be empty, had learned to mistrust the malevolent informant and to look for the object in the other box. If this first hypothesis is correct, children should revise their strategy in the second phase of the experiment: being told that the second informant using the unfamiliar communicative device is benevolent, they should then look for the object in the box indicated by the unfamiliar communicative device.

Alternatively, children may have kept trusting the first informant even though they were told he was mean. They may then have learned to avoid the box indicated by the unfamiliar communicative device, not because of distrust, but because they revised their initial interpretation and inferred that what the unfamiliar communicative device meant was that the box that it indicated was empty. If this second hypothesis is correct, children who had trusted the first informant described as “mean” should a fortiori trust the second informant described as “nice” and should continue to avoid the box indicated by the unfamiliar communicative device.

We also tested a different group of children in a baseline trust condition in order to make sure that the initial phase in which the informant was "mean" played a role in guiding children's interpretation of the unfamiliar communicative device's meaning. This baseline trust condition was identical to the test condition except that it only included the second phase of the experiment, in which the informant was characterized as nice and willing to help.

## Study 1

### Methods

#### Ethics statement

This research was approved by the institutional review board of the doctoral school ED3C (Ecole Normale Supérieure, Ecole des Hautes Etudes en Sciences Sociales, University Paris VI) and by the regional board of schools (Inspection Académique du Tarn). It was conducted in accordance with the ethical guidelines of the French National Research Center (CNRS). The participants’ parents provided written informed consent.

#### Participants

Three- to four-year-olds (*n* = 25; *M* = 4;1, range 3;1 to 4;11) were enrolled in the test condition. An additional group of children participated in the baseline trust condition (*n* = 12, *M* = 4;0, range 3;0 to 4;9). Six additional subjects were tested but excluded from data analysis because they failed to answer control questions more than three times (ages 3;2 and 3;3) or because they became fussy or uncooperative (ages 3;1, 3;5, 4;0 and 4;5). In Studies 1 and 2, no data on the ethnic origins of children were collected. In each school, all children of the target age group whose parents gave informed consent were tested, thus explaining sample size variations across age groups. The resulting sample sizes were comparable to those of similar previous studies [14, 27].

#### Settings and materials

Children were tested in their school, in a quiet room adjacent to their classroom. In all studies, the experimenter faced the child across a small table. Two boxes of different colors were placed at the center of the table, at equal distance of the participant. All the boxes were opaque and had lids, so that the child could not see what was in the boxes once they were closed. In each trial, a reward (a woolen pompom) was hidden in one of the boxes. To prevent carry-over effects, a different pair of boxes was used for each trial. Two different animal puppets (a cow and a frog) were used as informants. Puppets were named using the noun referring to their species (e.g. the frog puppet was referred to as ‘‘the frog”). Puppets used a marker (a 4x4cm white paper square) in order to communicate. The marker was referred to as ‘‘the piece of paper”.

#### Test condition

Before the experiment started, the experimenter showed the boxes, the pompom and the puppets to the participant. He also explained that he was going to hide the pompom in one of the two boxes and that the aim of the game was to find out where the pompom was. At the beginning of each trial, the experimenter opened the boxes and he asked the participant to turn round. He placed the pompom in one of the boxes, before closing them loudly. The child was then invited to look again with the remark: ‘‘That’s it! I have hidden the pompom!”. Whether the pompom was hidden in the box located on the right or left side of the table was randomized across trials. Similarly, for each pair of boxes, the box in which the pompom was placed was randomized across trials. For each trial, the puppet that acted as an informant was placed at the center of the table, behind the boxes.

Mean informant trials. The first phase of the test condition consisted of ten trials where the puppet that acted as an informant was described as “mean”. Each trial started with a characterization phase, that ensured that participants encoded information about the puppet’s meanness and lack of cooperativeness. During this initial characterization phase, the experimenter said: “Pay attention, the [name of the puppet, e.g. frog] is mean! It does not want you to find the pompom”. The experimenter then asked two control questions while pointing towards the puppet that acted as an informant: “Is it mean?” (control question 1) and: “Does it want you to find the pompom?” (control question 2). Children were corrected each time they failed to answer a control question. We excluded from the data analysis participants who failed to answer control questions appropriately more than three times during the experiment.

Once the child had answered the control questions, the experimenter explained: “Now the [name of the puppet, e.g. frog] is going to do something”. The experimenter manipulated the puppet so that it seemed to lift the marker before placing it on one of the boxes. The puppet that was said to be “mean” always placed the marker on the empty box. The experimenter then asked the test question: “Where is the pompom?”. The participants could answer the test question verbally or by pointing. At the end of the first trial, children received no information about the real location of the pompom. The experimenter simply said: “Let’s play again!” before swapping the boxes used during the first trial with a new pair of boxes. Children thus first participated in two “pre-training trials” with the mean puppet before receiving any feedback. We decided to have two pre-training trials in our Study (instead of one) in order to have more data about the behavior of participants before they received any feedback. From the end of the second trial on and for the eight subsequent trials, the experimenter gave feedback to the child by opening the boxes at the end of each trial, revealing the location of the pompom. The marker and the pompom were then placed at the center of the table, and memory prompts were given to the child: “Where was the pompom?” (memory prompt 1) “And where was the piece of paper?” (memory prompt 2). We assessed participants’ performance during the two first mean informant trials (pre-training trials); and during the two last mean informant trials (post-training trials). After the tenth mean informant trial, the experimenter said: “Now we are going to play with another animal!”. He replaced the “mean” puppet with a new one (e.g. a cow), and the nice informant trials started.

Nice informant trials. The second phase of the experiment consisted of two trials where the puppet that acted as an informant was described as “nice.” The trials involving the nice puppet unfolded just as the trials involving the mean puppet, with two exceptions. First, during the characterization phase occurring at the beginning of each trial, the experimenter conveyed that the puppet was benevolent and helpful by saying: “Pay attention, the [name of the puppet, e.g. cow] is nice! It wants you to find the pompom.” The control questions that followed were the same as in the mean informant trials (control question 1: “Is it mean?”; control question 2: “Does it want you to find the pompom?”). Second, children received no feedback about the actual location of the pompom at the end of each of the two nice informant trials. The type of puppet used to impersonate the “mean” or the “nice” informant—frog or cow—was randomized across subjects.

#### Baseline trust condition

The baseline trust condition was identical to the test condition, except that children did not participate in trials involving the “mean” puppet. Participants enrolled in this condition only participated in the presentation of the game, directly followed by two nice informant trials whose procedure was the same as in the test condition.

#### Scoring and data analysis

Data were coded online by the experimenter (as in previous studies using similar tasks [11, 13–14, 27–30]. In the test condition, the children’s performances were assessed at three stages: during the pre-training trials (the first two mean informant trials), that occurred before children received feedback about the location of the reward; during the post-training trials, i.e., on the last two mean informant trials (the ninth and the tenth trials); and during the nice informant trials that occurred at the very end of the procedure of the test condition. Statistics were computed over the percentage of trials in which the participants selected the box on which the marker was placed for each stage of the test condition (pre-training, post-training, and nice informant trials), and for the nice informant trials of the baseline condition. All the statistical tests reported in this paper are two-tailed. Given the discontinuous nature of our data, we used only non-parametric statistics.

### Results and discussion

**Fig 1:**
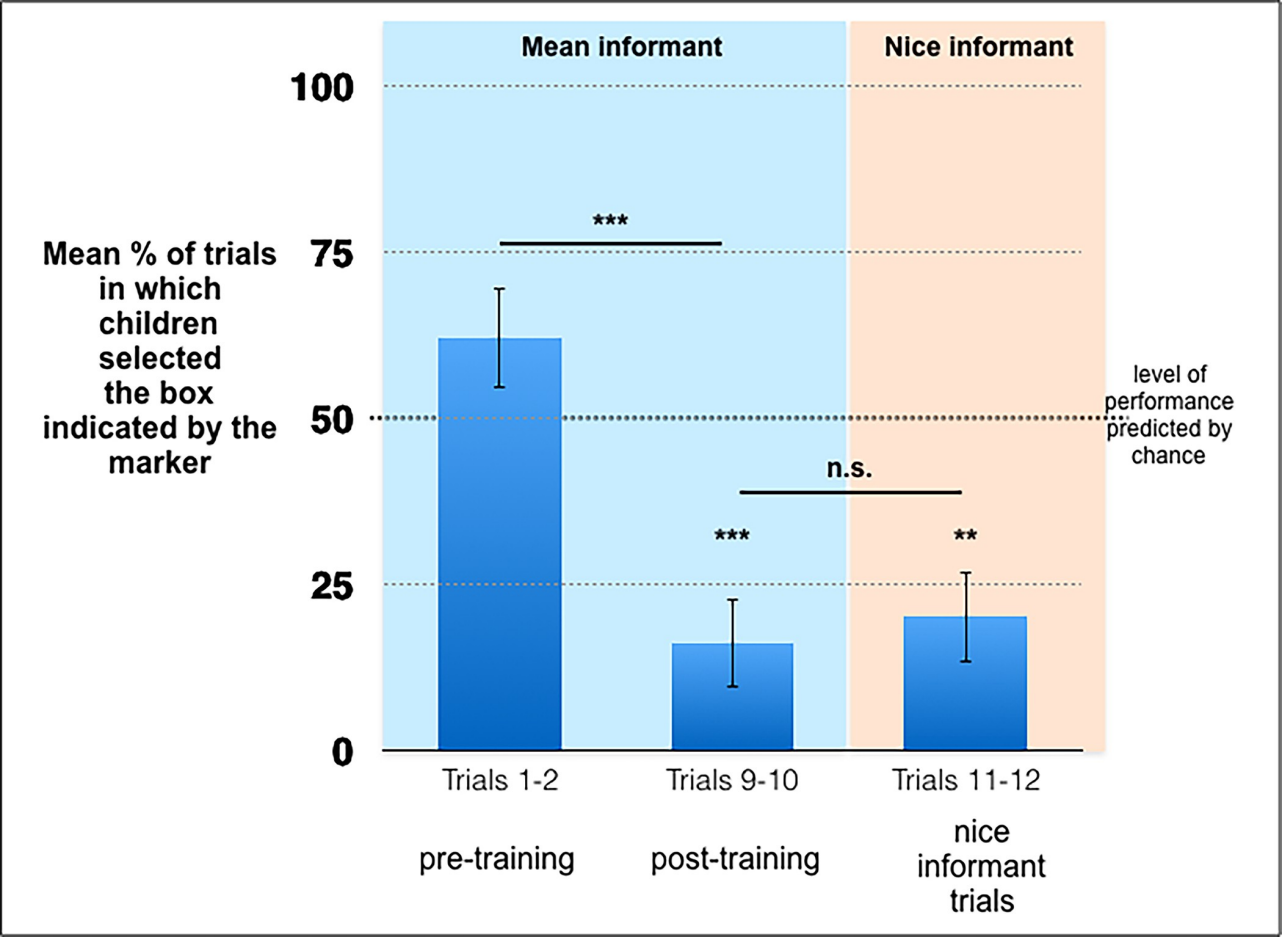
Average percentage of trials in which children selected the box indicated by the marker per phase of the test condition in Study 1 (SEM) (*: *p* < .05, **: *p* < .01, ***: *p* < .001).

Pre-training trials (Mean informant trials 1 and 2). As Fig 1 shows, the average percentage of the pre-training trials in which the participants selected the box on which the marker was placed did not differ significantly from what could be predicted by chance (*M* = 62%, *SD* = 38.16, *W* = 93.5, *p* = .13, one-sample Wilcoxon signed-rank test). Thus, before the participants received feedback about the location of the reward, they did not systematically avoid the box on which the marker was placed.

Post-training trials (Mean informant trials 9 and 10). In contrast, the average percentage of the post-training trials in which participants selected the box indicated by the marker was significantly lower from what could be predicted by chance (*M* = 16%, *SD* = 33.82, *W* = 36, *p* < .001, one-sample Wilcoxon signed-rank test). The participants were significantly less likely to select the box indicated by the marker during the post-training trials than during the pre-training trials (*W* = 166, *p* < .001, Wilcoxon signed-rank test for matched pairs). Thus, receiving feedback about the real location of the reward increased the participants’ tendency to avoid the box indicated by the marker.

Nice Informant Trials (Trials 11 and 12). The average percentage of the nice informant trials in which the participants selected the box indicated by the marker was significantly lower from what could be predicted by chance (*M* = 20%, *SD* = 34.64, *W* = 33, *p* = .001, one-sample Wilcoxon signed-rank test). The participants did not select the box indicated by the marker significantly more often during the nice informant trials than during the post-training mean informant trials (*W* = 17.5, *p* = .588, Wilcoxon signed-rank test for matched pairs). Subsequently, in the test condition, participants continued to avoid the box on which the marker was placed even when the malevolent informant was replaced by a benevolent one.

#### Baseline trust condition

In the baseline trust condition, the participants did not select the box indicated by the marker more often than predicted by chance (*M* = 62.5%, *SD* = 36.08, *W* = 20, *p* = .25, one-sample Wilcoxon signed-rank test). The participants’ tendency to select the box indicated by the marker was not significantly higher in the baseline trust condition than in the pre-training trials of the test condition (*U* = 150, *p* = .98, Mann-Whitney U test). In contrast, the participants were significantly more likely to select the box indicated by the marker in the baseline trust condition than during the post-training trials of the test condition (*U* = 242.5, *p* = .003, Mann-Whitney U test), or during the nice informant trials of the test condition (*U* = 235.5, *p* = .006, Mann-Whitney U test).

In order to focus on the role of learning on performance, we re-analyzed separately the performance of the participants enrolled in the test condition who selected the box indicated by the mean informant less often during the post-training trials than during the pre-training trials. This selection procedure allowed us to focus on the “learners”, i.e., on the participants who benefitted from receiving feedback about the real location of the reward (*n* = 17). The average percentage of the pre-training trials in which the learners selected the box indicated by the marker was significantly higher than predicted by chance (*M* = 79.41%, *SD* = 24.61, *W* = 55, *p* = .002, one-sample Wilcoxon signed-rank test). In contrast, the average percentage of the post-training trials in which the learners selected the box indicated by the marker was significantly lower than predicted by chance (*M* = 5.88%, *SD* = 16.11, *W* = 0, *p* < .001, one-sample Wilcoxon signed-rank test). The average percentage of the nice informant trials in which the learners selected the box indicated by the marker was significantly lower than predicted by chance (*M* = 17.65%, *SD* = 34.05, *W* = 16, *p* = .004, one-sample Wilcoxon signed-rank test). The learners did not select the box indicated by the marker significantly more often during the nice informant trials than during post-training trials (*W* = 12.5, *p* = .20, Wilcoxon signed-rank test for matched pairs). In addition, the learners’ tendency to select the box indicated by the marker was significantly higher in the baseline trust condition than during the nice informant trials of the test condition (*U* = 163, *p* = .007, Mann-Whitney U test). In short, the analyses performed on the subgroup of “learners” confirmed the analyses performed on the whole group of participants.

In the test condition of Study 1, participants learned to avoid the box on which a mean informant placed a marker. Yet, they did not revise this strategy when subsequently, a nice informant used the marker. Still, in Study 1, participants had to discover the function of the marker (communicative) and its meaning (indicating the location of the pompom). The fact that some children misinterpreted the function or the meaning of the marker may be a mere result of its high ambiguity. Indeed, the fact that children did not follow the marker more often than predicted by chance in the baseline condition opens the possibility that the participants may have found it difficult to recognize the communicative nature of the marker. Additionally, the initial phase involving the mean informant was fairly long in Study 1. Maybe some children initially displayed vigilance towards deceptive intents but later engaged in blind avoidance of the marker, for example, because of boredom. To control for these limitations, we designed a second Study with a shorter initial phase involving the mean informant, and in which the informant used a device that was less ambiguous than the marker. In this second Study, the informants communicated using an arrow, i.e. a communicative device whose meaning is conventionalized to some extent. Furthermore, the communicative function of the arrow was demonstrated at the beginning of the Study.

## Study 2

### Methods

#### Participants

Three- to four-year-olds (*n* = 28; *M* = 4;1, range 3;0 to 4;9) were enrolled in the test condition. An additional group of children participated in the baseline trust condition (*n* = 16, *M* = 4;1, range 3;2 to 4;9). Six additional subjects were tested but excluded from data analysis because they failed to answer control questions more than three times (ages 3;0, 3;11 and 4;1) or because they were unresponsive (ages 2;9, 3;0 and 3;0). The recruitment procedure was the same as in Study 1.

#### Settings and materials

The materials of Study 2 were the same as those of Study 1, except that the informants manipulated a 2D cardboard arrow instead of a marker to communicate the location of the reward. The arrow was laid horizontally on the testing table and it was placed at equidistance of the two boxes that could contain the reward. It was possible to rotate the arrow to point towards one of the boxes.

#### Test condition

The procedure of the test condition was identical in Study 2 and in Study 1, with the following exceptions. First, the experimenter demonstrated the communicative nature of the arrow at the very beginning of the experiment. After placing two boxes, the arrow and the pompom on the testing table, the experimenter said: “We are going to play a game with two boxes, a pompom, and an arrow.”. Then, the experimenter explained: “With the arrow, I can show a box …”. Next, the experimenter rotated the arrow towards one of the boxes while asking the first prompt question: “Which box am I showing?”. Then, the experimenter rotated the arrow towards the alternate box, while asking the second prompt question: “And now …, which box am I showing?” When participants did not answer appropriately the prompt questions, they were corrected. Second, in Study 2, the mean informant trials and the nice informant trials unfolded exactly as in Study 1 except that the puppets communicated by rotating the arrow towards one of the boxes. Third, children participated in only four consecutive mean informant trials. Children first participated in two pre-training trials in which the mean puppet manipulated the arrow. At the end of the first pre-training trial, children received no feedback about the real location of the pompom. Children thus first participated in the two pre-training trials with the mean puppet before receiving any feedback. From the end of the second trial on and for the two subsequent trials, the experimenter gave feedback to the child by opening the boxes at the end of each trial, revealing the location of the pompom. The arrow and the pompom were then placed at the center of the table, and memory prompts were given to the child: “Where was the pompom?” (memory prompt 1) “And which box did the arrow show?” (memory prompt 2). The mean informant trials ended with the fourth trial (thus, the third and fourth trials were the post-training trials). The transition from the post-training trials (trials 3 and 4) to the nice informant trials (trials 5 and 6) unfolded as in Study 1. The participants were enrolled in two nice informant trials. As in Study 1, the type of puppet used to impersonate the “mean” or the “nice” informant—frog or cow—was randomized across subjects.

#### Baseline trust condition

As in Study 1, the baseline trust condition was identical to the test condition, except that children did not participate in the trials involving the “mean” puppet. They only participated in the presentation of the game, directly followed two trials that unfolded like the nice informant trials of the test condition.

#### Scoring and data analysis

As in Study 1, statistics were computed over the percentage of trials in which the participants selected the box indicated by the unfamiliar communicative device (the arrow in Study 2) for each stage of the test condition (pre-training, post-training, and nice informant trials), and for the nice informant trials of the baseline condition.

### Results and discussion

#### Test condition

**Fig 2:**
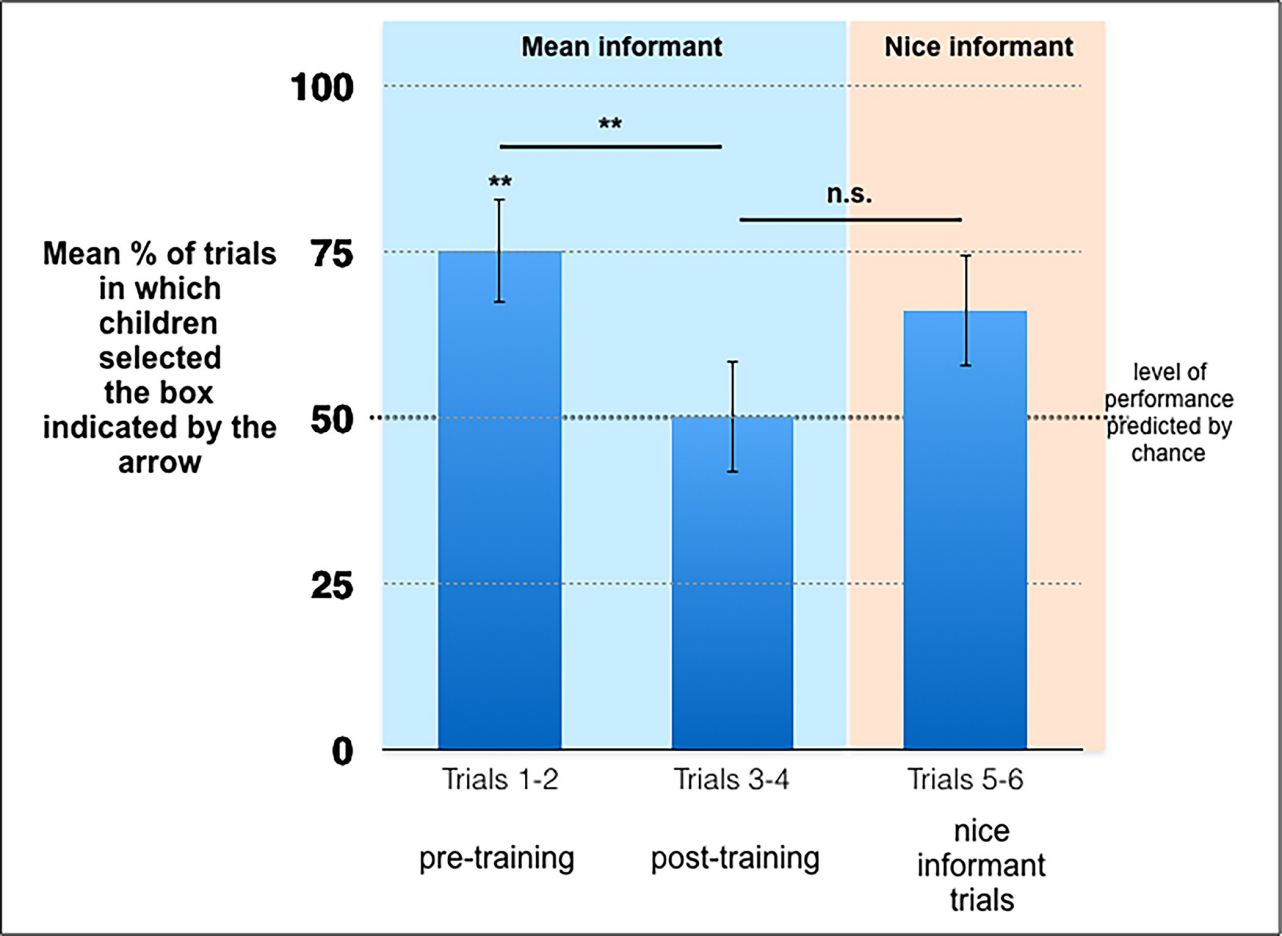
Average percentage of trials in which children selected the box indicated by the arrow per phase of the test condition in Study 2 (SEM) (*: *p* < .05, **: *p* < .01, ***: *p* < .001).

Pre-training trials (Trials 1 and 2). As Fig 2 shows, in Study 2 the average percentage of the pre-training trials in which the participants selected the box indicated by the arrow was significantly higher than predicted by chance (*M* = 75%, *SD* = 41.19, *W* = 270, *p* = .006, one-sample Wilcoxon signed-rank test). Furthermore, 26 out of 28 participants kept using the same strategy during the first two trials of the test condition—either selecting the box indicated by the arrow twice or avoiding the box that was indicated by the arrow twice (*p* < .001, two-choice binomial test). These results reveal that during the pre-training trials, the participants of Study 2 expected to find the reward in the box indicated by the arrow.

Post-training trials (Trials 3 and 4). The average percentage of the post-training trials in which the participants of Study 2 selected the box indicated by the arrow did not differ significantly from what could be predicted by chance (*M* = 50%, *SD* = 44.32, *W* = 126.5, *p* = 1, one-sample Wilcoxon signed-rank test). As in Study 1, the participants were significantly less likely to select the box indicated by the arrow during the post-training trials than during the pre-training trials (*W* = 82, *p* = .009, Wilcoxon signed-rank test for matched pairs). Thus, receiving feedback about the real location of the reward increased the participants’ tendency to avoid the box indicated by the arrow. In the post-training trials of Study 2, the participants’ tendency to select the box indicated by the arrow did not differ from chance. Thus, we performed complementary analyses of individual strategies to assess whether children simply stopped paying attention to the arrow, or whether they used it in a systematic fashion. These analyses indicated that in Study 2, 22 out of 28 participants kept using the same strategy during the two post-training trials of the test condition (*p* = .004, two-choice binomial test)—either selecting the box indicated by the arrow twice (*n* = 11) or avoiding the box that was indicated by the arrow twice (*n* = 11). These results rule out the possibility that participants simply learned to ignore the arrow. They indicate that the children used the arrow in a systematic fashion during the post-training trials.

Nice Informant Trials (Trials 5 and 6). In Study 2, the average percentage of the nice informant trials in which the participants selected the box indicated by the arrow tended to be higher than predicted by chance (*M* = 66.07%, *SD* = 45.25, *W* = 252, *p* = .086, one-sample Wilcoxon signed-rank test). The participants did not select the box indicated by the arrow significantly more often during the nice informant trials than during the post-training trials (*W* = 51.50, *p* = .1, Wilcoxon signed-rank test for matched pairs).

#### Baseline trust condition

In the baseline trust condition, participants selected the box indicated by the arrow more often than predicted by chance (*M* = 93.75%, *SD* = 17.08, *W* = 105, *p* < .001, one-sample Wilcoxon signed-rank test). The participants’ tendency to select the box indicated by the arrow was not significantly higher in the baseline trust condition than in the pre-training trials of the test condition (*U* = 266, *p* = .312, Mann-Whitney U test). In contrast, in Study 2, the participants’ tendency to select the box indicated by the arrow tended to be higher in the baseline trust condition than during the post-training trials of the test condition (*U* = 342, *p* = .003, Mann-Whitney U test), and during the nice informant trials of the test condition (*U* = 292, *p* = .099, Mann-Whitney U test). These results confirm that the children learned to avoid the box indicated by the arrow during the mean informant trials.

In order to focus on the role of learning on performance, we re-analyzed separately the performance of the “learners”, i.e., the participants enrolled in the test condition who selected the box indicated by the mean informant less often during the post-training trials than during the pre-training trials (*n* = 11). In Study 2, the average percentage of the pre-training trials in which the learners selected the box indicated by the arrow was significantly higher than predicted by chance (*M* = 95.45%, *SD* = 14.37, *W* = 55, *p* = .001, one-sample Wilcoxon signed-rank test). In contrast, the average percentage of the post-training trials in which Study 2’s learners selected the box indicated by the arrow was significantly lower than predicted by chance (*M* = 22.73%, *SD* = 26.11, *W* = 0, *p* = .014, one-sample Wilcoxon signed-rank test). Furthermore, the average percentage of the nice informant trials in which Study 2’s learners selected the box indicated by the arrow was significantly lower than predicted by chance (*M* = 18.18%, *SD* = 32.14, *W* = 5, *p* = .02, one-sample Wilcoxon signed-rank test). Thus, the learners continued to avoid the box indicated by the arrow even when it was manipulated by a benevolent informant. In addition, Study 2’s learners did not select the box indicated by the arrow significantly more often during the nice informant trials than during post-training trials (*W* = 4, *p* = .77, Wilcoxon signed-rank test for matched pairs). Moreover, Study 2’s learners’ tendency to select the box indicated by the arrow was significantly higher in the baseline trust condition than during the nice informant trials of the test condition (*U* = 165, *p* < .001, Mann-Whitney U test). In short, as in Study 1, the analyses performed on the subgroup of “learners” confirmed the analyses performed on the whole group of participants in Study 2.

The data of Study 2 confirm the pattern of results observed in Study 1. In Study 2, unlike in Study 1, the communicative nature of the unfamiliar signal was made explicit to the participants. Accordingly, they used the unfamiliar signal (the arrow) to locate the toy prior to receiving any feedback about the reward’s location, both when it was used by a benevolent informant (in the baseline trust condition) or by a malevolent informant (in the pre-training trials). Crucially, as in Study 1, Study 2’s participants did not adjust their use of the unfamiliar communicative device to the benevolence of the informant. In Study 2’s test condition, the children who learned to avoid the arrow during the mean informant trials did not change their strategy when presented with a benevolent informant, and they kept avoiding the location indicated by the arrow.

## General discussion

Prior to four to five years of age, children keep trusting a misleading informant using a familiar communicative device such as pointing or verbal testimony over many repeated trials [11, 13–16, 27]. In contrast, when the communicative device is less familiar, three- to four-year-olds learn to avoid the location that it indicates. In previous studies, this phenomenon has been interpreted as evidence that what is conveyed by unfamiliar devices is accepted less rigidly than what is communicated by familiar devices such as pointing or verbal testimony [14, 27]. Our studies show that this is not the only interpretation possible.

According to the alternative interpretation suggested by our studies, it is neither that 3-year-olds trust other people in all respects—they understand that mean people do bad things—nor that what they trust are specific and familiar communicative devices. Rather, children view overt, ostensive communication in general as an intrinsically cooperative, ignore the possibility of deception, and hence trust people qua communicators, whether or not these people are nice or mean otherwise. On the basis of this trust in communication, children may reinterpret an unfamiliar communicative device repeatedly indicating a box that turned out to be empty as in fact denoting the empty box.

More specifically, in our Studies, once they started receiving feedback about the location of the pompom, children reduced their tendency to select the box indicated by the unfamiliar communicative device. This, so far, is little more than a conceptual replication of [14, 27] and is merely consistent with our alternative hypothesis. What support this alternative hypothesis is the fact that participants didn’t revise this strategy when the “mean” informant was replaced by a “nice” one as they should have if they trusted this second informant and interpreted the unfamiliar signal as indicating the location of the pompom. Furthermore, in Studies 1 and 2, children’s likelihood of avoiding the location indicated by the unfamiliar communicative device used by a “nice” informant tended to be significantly stronger in the test condition than in the baseline trust condition. This last result was most evident in the sub-group of “learners”, i.e., in the subset of participants who selected the box indicated by the novel signal less often after receiving feedback about the location of the reward (during the post-test trials), than at the beginning of the experiment (during the pre-test trials).

These results are inconsistent with the hypothesis that repeated trials in the initial training phase had helped children to recognize that the uncooperative “mean” informant intended to deceive them. If this hypothesis had been correct, children should have come back to their initial level of baseline trust when presented with a “nice” and cooperative informant. These results are also inconsistent with the hypothesis that what children distrusted was not so much the mean communicator as the unfamiliar communicative device and that they simply learned to ignore this device. If children had treated the unfamiliar communicative devices as uninformative and irrelevant, they should have selected one of the boxes randomly. Rather, they relied systematically on the marker to locate the empty box in Study 1. In the post-training trials of Study 2, the participants used one of two strategies: some children systematically avoided the box that the arrow was pointing to, while other children systematically selected it.

In other words, these results suggest that training had caused children to use the unfamiliar communicative device as a signal reliably indicating the location of the empty box. This “smart trusting hypothesis” postulates that when signals have no predetermined interpretation, young preschoolers can without difficulties interpret them so as to fit their expectation that communication is honest and helpful. Such smart trusting may well play an essential role in helping children infer the meaning of novel words and of other unfamiliar signals. By contrast, reinterpreting a signal whose meaning has been well-established (such as pointing) should be much harder.

The smart trusting hypothesis is compatible with the view that trust in communication comes with the recognition of a behavior as intended to communicate, whatever the nature of the behavior employed. Still, familiarity with communicative means may be important in two other ways. First, the use of familiar signals makes it quite clear that communication is intended whereas unfamiliar forms of communication may not be as easily recognized as communicative. Familiarity with the means employed by the communicator may therefore indirectly increase trust by making it clear that communication is intended in the first place. Indeed, there is evidence showing that children rely more on an unfamiliar signal when it is accompanied by cues indicating an intention to communicate [13, 31]. Such cues do not turn unfamiliar signals into familiar ones. What they do is it makes it clearer that the unfamiliar behavior is used to communicate. Second, even when the intention to communicate has been established, familiar signals may still be harder to re-interpret, quite simply because their meaning has been well-established in the past.

One methodological caveat (identified by a reviewer) from our study is that in the test condition children might learn to inflexibly avoid the box indicated by the novel communicative device. This “inflexible learning” interpretation postulates that children first learned to select the box that was not indicated by the unfamiliar signal, and then did not manage to adjust flexibly their strategy to the change of informants. It should be noted that during the mean informant trials, a large subset of participants learned to systematically avoid the box indicated by the marker (Study 1), or by the arrow (Study 2). Thus, as a group, children did demonstrate the capacity to change their strategy in our Studies. Yet, we cannot entirely rule out that if children participated in more trials with the nice informants they would at some point shift strategy. Crucially, the inflexible learning hypothesis implies that children’s avoidance of the location indicated by an unfamiliar signal is not a demonstration of vigilance towards the potential falsity of what the unfamiliar signal conveys. Thus, regardless of whether our smart trusting hypothesis or the alternative inflexible learning interpretation is true, our study shows that the finding in earlier studies [14, 27] that children can learn to avoid the location indicated by an unfamiliar communicative signal cannot be taken as unambiguous evidence for distrust of such signals.

Our data do not tell why children do not learn to reinterpret familiar signals that are repeatedly misleading [11, 13–14, 27], but this is not much of a puzzle. Young children may simply not revise their interpretation of familiar signals because past experience makes them quite confident about the meaning of these signals (just like adults would not automatically assume that familiar words are used with a different meaning when, taken literally, they convey a misleading message).

Children tested in our study were made to pay attention to the benevolence or malevolence of the informants. Yet, they did not use this information to adjust their acceptance of communicated information. These results are all the more remarkable given that children as young as three properly understand the terms “nice” and “mean” when selecting informants: they prefer to endorse and request testimony from characters that have been said to be “nice” rather than “mean” [12, 32–33]. More generally, by the age of four—the mean age of our participants—children are sensitive to the accuracy with which the terms “nice” and “mean” are employed [34], and they use these terms to make consistent behavioral predictions in a variety of situations [35]. Furthermore, we ensured that children paid attention to what was said to them about the cooperativeness of informants by asking control questions about the benevolence of the informants at the beginning of each trial (“Is it mean?” and “Does it want you to find the pompom?”). Participants who failed to answer correctly these control questions more than three times during the experiment were excluded from the analysis.

Yet, children did not calibrate their reliance on the unfamiliar signal depending on the benevolence of informants. This pattern of results is compatible with the view that before four to five years of age, children have difficulties either with (i) recognizing that malevolent individuals might use deception and lies to achieve their aims, or with (ii) acting upon the knowledge that malevolent informants might use deception (e.g., for executive reasons). Children's inability to flexibly integrate information about the benevolence or malevolence of informants could explain their difficulty with remaining vigilant towards deception from malevolent or uncooperative agents.

## Supporting information

S1 Data Full dataset. (XLS)

## References

[1] Lewis D. Convention. Cambridge: Harvard University Press; 1969.

[2] Coady CAJ. Testimony: A philosophical study. 1^st^ ed. Oxford: Oxford University Press; 1992.

[3] Harris PL, Koenig MA. Trust in testimony: How children learn about science and religion. Child Dev. 2006;77(3): 505–524. 10.1111/j.1467-8624.2006.00886.x 16686784

[4] Clément F. To Trust or not to Trust? Children’s Social Epistemology. Rev Phil Psychol. 2010;1(4): 531–549.

[5] Harris PL, Corriveau KH. Young children’s selective trust in informants. Phil T Roy Soc B: Biol Sci. 2011;366(1567):1179–1187. 10.1098/rstb.2010.0321 PMC3049091 21357240

[6] Mills CM. Knowing when to doubt: developing a critical stance when learning from others. Dev Psychol. 2013;49(3): 404–418. 10.1037/a0029500 22889395 PMC3810952

[7] Nurmsoo E, Robinson EJ, Butterfill SA. Children’s Selective Learning from Others. Rev Phil Psychol. 2010;1(4): 551–561.

[8] Clément F, Koenig M, Harris P. The ontogenesis of trust. Mind Lang. 2004;19(4): 360–379.

[9] Ma L, Ganea PA. Dealing with conflicting information: young children’s reliance on what they see versus what they are told. Dev Sci. 2010;13(1): 151–160. 10.1111/j.1467-7687.2009.00878.x 20121871

[10] Robinson EJ, Whitcombe EL Children's suggestibility in relation to their understanding about sources of knowledge. Child Dev. 2003;74(1): 48–62. 10.1111/1467-8624.t01-1-00520 12625435

[11] Mascaro O, Sperber D The moral, epistemic, and mindreading components of children’s vigilance towards deception. Cognition 2009;112(3): 367–380. 10.1016/j.cognition.2009.05.012 19540473

[12] Mascaro O, Morin O. Epistemology for Beginners: Two-to Five-Year-Old Children's Representation of Falsity. PloS one 2015;10(10): e0140658 10.1371/journal.pone.0140658 26484675 PMC4618725

[13] Heyman GD, Sritanyaratana L, Vanderbilt KE. Young children's trust in overtly misleading advice. Cog Sci. 2013;37(4): 646–667. 10.1111/cogs.12020 PMC8063353 23294130

[14] Jaswal VK, Croft AC, Setia AR, Cole CA. Young children have a specific, highly robust bias to trust testimony. Psychol Sci. 2010; 21(10): 1541 10.1177/0956797610383438 20855905 PMC3507998

[15] Vanderbilt KE, Liu D, Heyman GD. The development of distrust. Child Dev. 2011;82(5): 1372–1380. 10.1111/j.1467-8624.2011.01629.x 21824130 PMC3169730

[16] Mascaro O, Morin O, Sperber D. Optimistic expectations about communication explain children's difficulties in hiding, lying, and mistrusting liars. J Child Lang. 2016;44(5): 1041–1064. 10.1017/S0305000916000350 27748210

[17] Grice P. Studies in the way of words. Cambridge, MA: Harvard University Press; 1989.

[18] Sperber D, Wilson D. Relevance: Communication and cognition. 2d ed Oxford: Blackwell; 1995.

[19] Origgi G. Is trust an epistemological notion? Episteme 2004;1(1): 61–72.

[20] Carey S, Bartlett E. Acquiring a single new word. Papers and Reports on Child Lang Dev. 1978;15: 17–29.

[21] Jaswal VK, Markman EM. Learning proper and common names in inferential versus ostensive contexts. Child Dev. 2001;72(3): 768–786. 10.1111/1467-8624.00314 11405581

[22] Gergely G, Bekkering H, Király I. Rational imitation in preverbal infants. Nature 2002;415(6873): 755–755. 10.1038/415755a 11845198

[23] Meltzoff AN. Understanding the intentions of others: Re-enactment of intended acts by 18-month-old children. Dev Psychol. 1995;31(5): 838–850. 10.1037/0012-1649.31.5.838 25147406 PMC4137788

[24] Csibra G, Gergely G. Natural pedagogy. Trends Cog Sci. 2009;13(4): 148–153. 10.1016/j.tics.2009.01.005 19285912

[25] Mascaro O, Morin O. Gullible’s travel: How honest and trustful children become vigilant communicators In: Einav S, Robinson E, editors. Trust and skepticism: Children's selective learning from testimony. Psychology Press; 2014 pp. 69–83.

[26] Palmquist CM, Burns HE, Jaswal VK. Pointing disrupts preschoolers’ ability to discriminate between knowledgeable and ignorant informants. Cog Dev. 2012;27(1): 54–63. 10.1016/j.cogdev.2011.07.002 PMC3256585 22247591

[27] Couillard NL, Woodward AL. Children’s comprehension of deceptive points. Br J Dev Psychol. 1999;17: 515–521.

[28] Tomasello M, Call J, Gluckman A. Comprehension of novel communicative signs by apes and human children. Child Dev. 1997;68(6): 1067–1080. 9418226

[29] Call J, Tomasello M. A nonverbal false belief task: The performance of children and great apes. Child Dev. 1999;70(2): 381–395. 10.1111/1467-8624.00028 10218261

[30] Vanderbilt KE, Heyman GD, Liu D. In the absence of conflicting testimony young children trust inaccurate informants. Dev Sci. 2014;17(3): 443–451. 10.1111/desc.12134 24444426

[31] Leekam SR, Solomon TL, Teoh YS. Adults’ social cues facilitate young children’s use of signs and symbols. Dev Sci. 2010;13(1): 108–119. 10.1111/j.1467-7687.2009.00862.x 20121867

[32] Landrum AR, Mills CM, Johnston AM. When do children trust the expert? Benevolence information influences children's trust more than expertise. Dev Sci. 2013;16(4): 622–638. 10.1111/desc.12059 23786479

[33] Lane J. D., Wellman H. M., & Gelman S. A. (2013). Informants' traits weigh heavily in young children's trust in testimony and in their epistemic inferences. Child Dev. 84(4), 1253–1268. 10.1111/cdev.12029 23240893 PMC3601569

[34] Boseovski JJ. Trust in testimony about strangers: Young children prefer reliable informants who make positive attributions. J Exp Child Psychol. 2012;111(3): 543–551. 10.1016/j.jecp.2011.10.008 22115450

[35] Liu D, Gelman SA, Wellman HM. Components of Young Children’s Trait Understanding: Behavior‐to‐Trait Inferences and Trait‐to‐Behavior Predictions. Child Dev. 2007;78(5): 1543–1558. 10.1111/j.1467-8624.2007.01082.x 17883447

